# ASC-emotion: A privacy-aware dataset for analysing emotional dysregulation and engagement in children with Autism^,^

**DOI:** 10.1016/j.mex.2026.104073

**Published:** 2026-07-27

**Authors:** Zakia Batool Turabee, David J. Brown, Mufti Mahmud, Andreas Oikonomou, Muhammad Arifur Rahman, Andrew Burton, Nicholas Shopland, Dawn Clarke, Fiona Gray

**Affiliations:** a Department of Computer Science, Nottingham Trent University, Nottingham, NG11 8NS, United Kingdom; b Autism Team, SEND Support Services, Education Division, Nottingham City Council, Nottingham, NG8 3BP, United Kingdom; c Information and Computer Science Department, King Fahd University of Petroleum and Minerals, Dhahran, 31261, Saudi Arabia; d SDAIA-KFUPM Joint Research Center for AI, King Fahd University of Petroleum and Minerals, Dhahran, 31261, Saudi Arabia; e Interdisciplinary Research Center for Biosystems and Machines, King Fahd University of Petroleum &amp; Minerals, Dhahran, 31261, Saudi Arabia

**Keywords:** Autism spectrum condition (ASC), Challenging behaviours, Facial expressions, Rumble moments, Emotional dysregulation, Engagement and arousal tracking, Camera and wearables

## Abstract

Predicting emotional dysregulation events in children with Autism is essential for timely mitigation of triggering events and prevention of further escalation of the situation. However, there is a scarcity of accessible and standarised datasets for use in AI-based research associated with challenging behaviours in children with ASC. To address this gap, we have curated a novel privacy-preserved dataset as part of an Erasmus+ funded project (AI-TOP-2020–1-UK01-KA201–079,167). The dataset was validated using three machine learning architectures which gave an accuracy of 96% in detecting the affective states related to learning in children with Autism. The key contributions of this study are:

1. Development of an Autism meltdown dataset exemplifies methodological rigor, advances an urgent clinical challenge through early detection and intervention, and enables broad impact by promoting reproducibility, benchmarking and translational health outcomes.

2. Implementation of privacy preserving measures addresses ethical concerns regarding the use of video data with this vulnerable population as part of the machine learning pipeline.

3. High levels of accuracy are demonstrated via empirical validation of the dataset through three machine learning models (BiLSTM, Graphical Neural Network – EdgeConv, PointCNN+LSTM) for detecting affective states related to learning and physiological arousal in children with Autism.

## Graphical abstract

**Figure:**
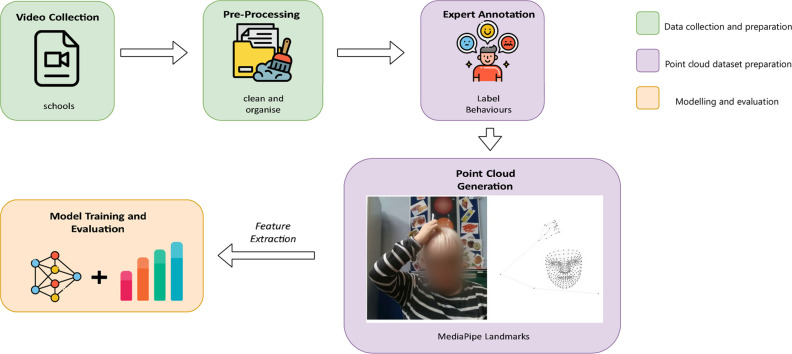

**Specifications table**

**Table:** 

**Subject area**	Computer Science
**More specific subject area**	Engagement and arousal (emotional dysregulation) tracking in children with Autism
**Name of your method**	Emotional dysregulation detection using a point cloud dataset generated from video data using MediaPipe
**Name and reference of original method**	None
**Resource availability**	Repository name: **Kaggle** Data identification number: **10.34740/kaggle/dsv/8,356,674** Direct URL to data: https://www.kaggle.com/datasets/zakiaturabee/asc-emotion

## Background

Autism Spectrum Condition (ASC) is classified as a neurodevelopmental disorder in the Diagnostic and Statistical Manual of Mental Disorders, 5th Edition (DSM-5) [1]. In the UK, it is estimated that more than one in every 100 people is Autistic, with at least 700,000 Autistic children and adults nationwide [2]. Early signs of ASC often include delayed language development, repetitive behavioural patterns and limited or no social engagement. Our research focuses on developing toolsets and platforms that use camera data and machine learning to assess classroom engagement and physiological or affective states, such as arousal and dysregulation, in children with ASC.

Given the heterogeneous nature of ASC, traits and behaviours associated with it exhibit very different patterns across individuals. Therefore, recognising and responding to children's emotional and behavioural cues in a classroom setting can be challenging. This challenge highlights the need to develop a multimodal system that monitors emotional dysregulation in students with ASC and alerts relevant caregivers or teaching staff to mitigate triggers for such events and prevent further escalation of the situation [3].

There is, however, a lack of available and accessible datasets that predict affective states related to learning (e.g., engagement, boredom, and frustration) and rumble moments in children with ASC. Despite our team making multiple attempts to contact the relevant authors, popular datasets such as 'Meltdown Crisis' and 'De Enigma' remained inaccessible. Furthermore, the majority of the studies which we reviewed rely on data from a limited number of participants within different contexts and settings. The populations from which these data are gathered are also highly skewed, such as the lack of details regarding the use of a control group, an imbalance in the ratio of girls to boys, or large age disparities among children. These issues in existing studies lessen their generalisability and increase the risk of overfitting [4,5].

In response, we have developed a dataset focused on learning behaviours and on the detection of emotional dysregulation events in children with ASC. An anonymised version of this dataset has been made publicly available, which serves as a valuable resource for research on behavioural patterns and emotional regulation in children with ASC. The dataset comprises data points extracted using Python’s MediaPipe library from classroom video recordings of children engaged in Continuous Performance Test (CPT) games. These videos were annotated using an observational behavioural checklist, developed in collaboration with Autism specialists from the Nottingham City Council Autism Team. The annotations provided labels used to segment video recordings as part of a supervised learning approach to understanding affective states related to and precursors of meltdown events (engagement, boredom, and frustration) [6].

This dataset will help in identifying early signs of challenging behaviour in children with ASC during classroom learning. Early detection supports timely intervention, reducing emotional dysregulation and classroom disruptions. Such outcomes will not only improve the learning environment but also foster more positive perceptions of Autism. To validate the dataset's usability, we applied a range of machine learning models; the results are discussed later in this paper.

Additionally, this dataset will be useful to the international research community interested in supporting the best educational experiences for children with Autism. It will help develop more effective teaching methods and support systems tailored to the needs of these children. Fig. 1, Fig. 2, Fig. 3, Fig. 4, Fig. 5, Fig. 6, Fig. 7, Fig. 8, Fig. 9, Fig. 10, Fig. 11, Fig. 12 and 14

**Fig. 1:**
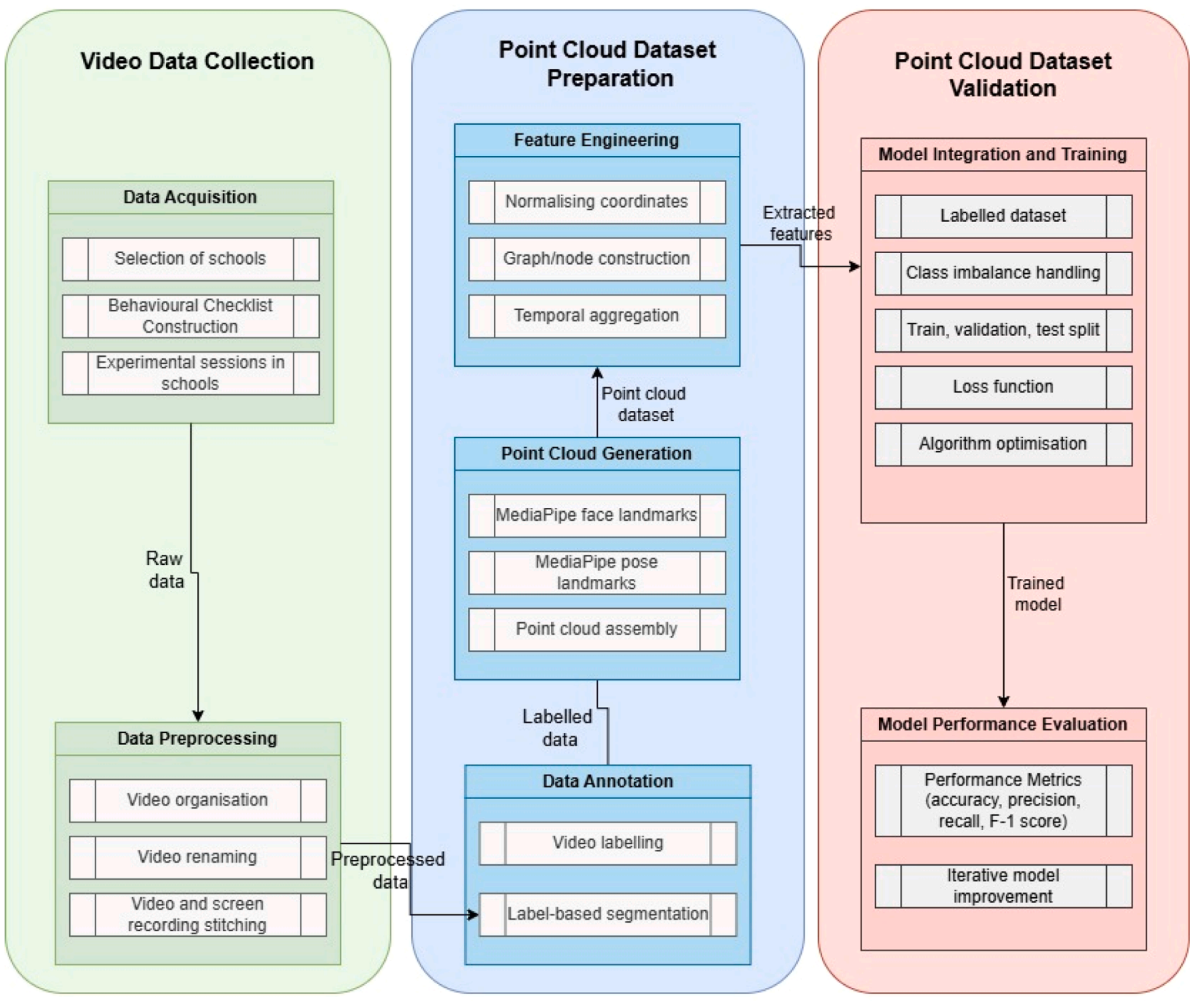
End-to-end pipeline for the ASC-Emotion dataset, illustrating the stages of video data collection, point cloud dataset preparation and model validation.

**Fig. 2:**
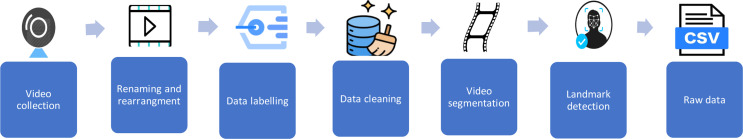
Block diagram of data collection process.

**Fig. 3:**
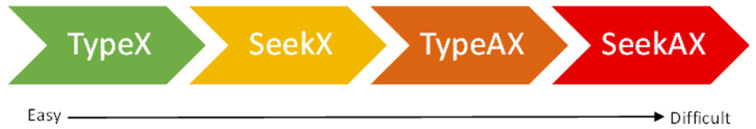
The progression of difficulty within the games.

**Fig. 4:**
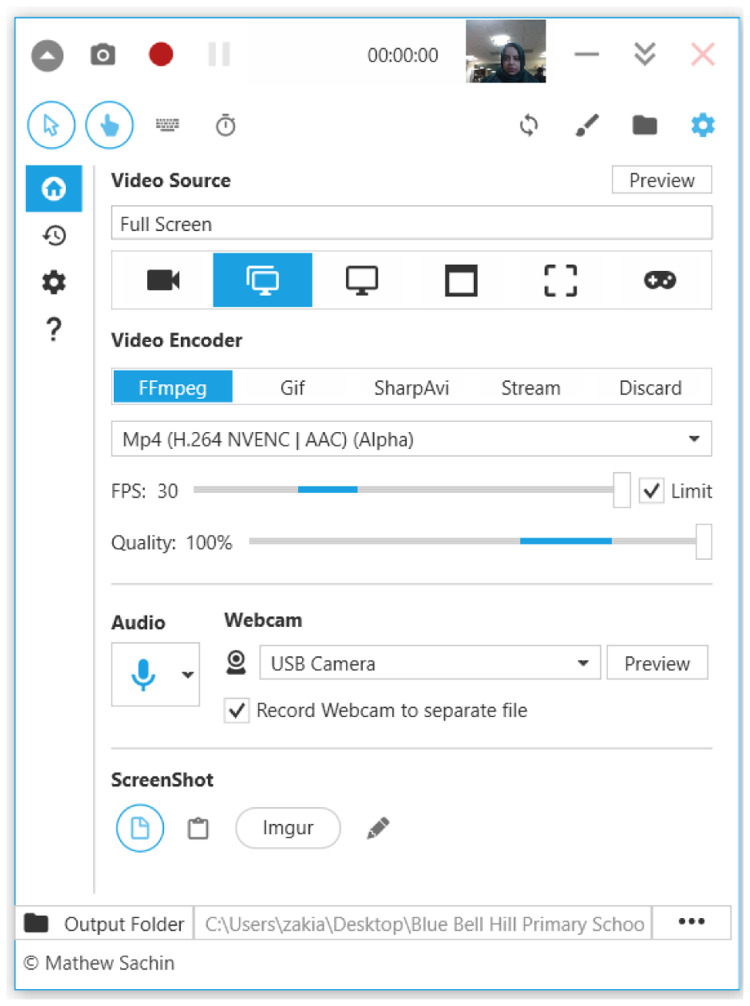
Captura software interface.

**Fig. 5:**
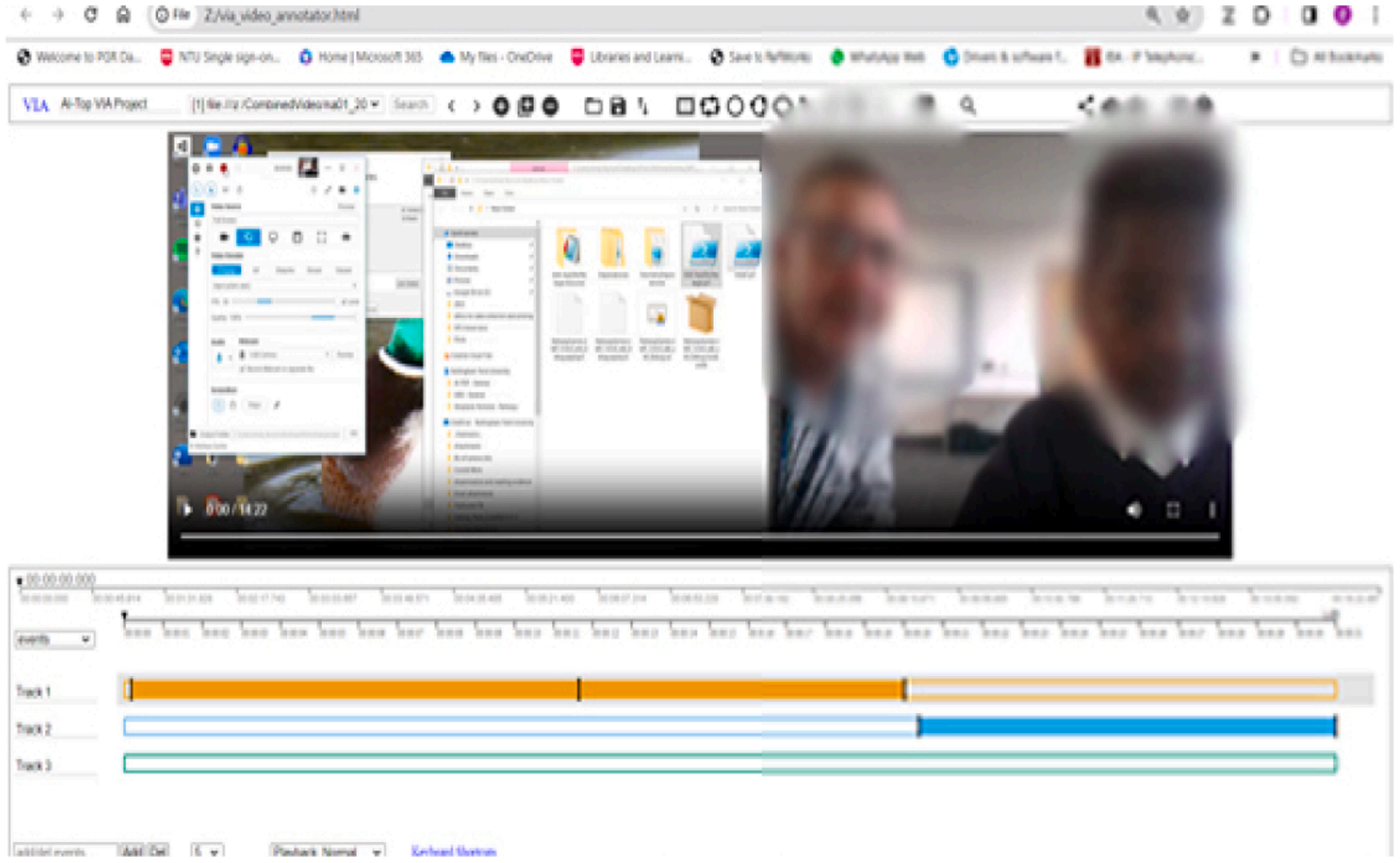
VIA software interface.

**Fig. 6:**
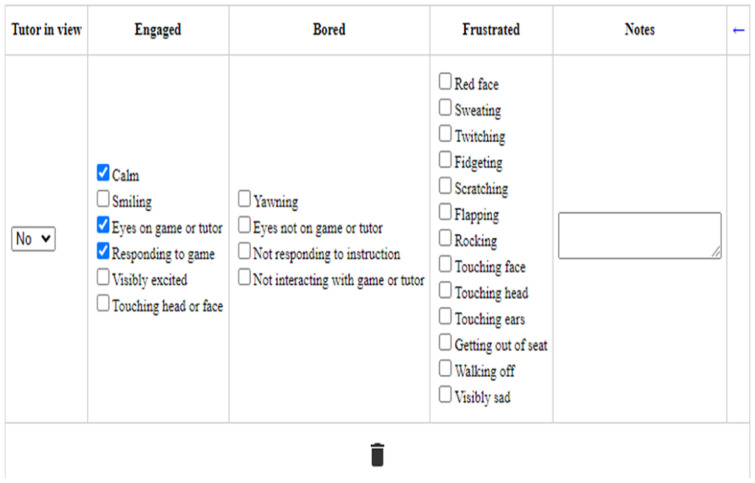
The checklists as they appear in the VIA software on adding an element to the observational timeline.

**Fig. 7:**
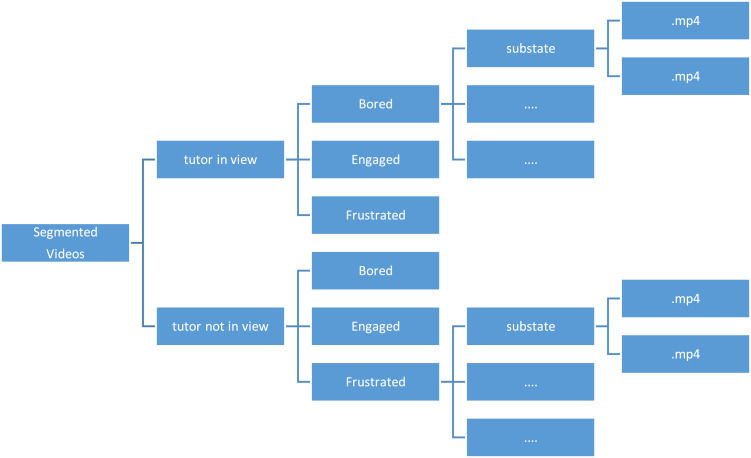
Hierarchy of initial video data folder on NTU's secure drive.

**Fig. 8:**
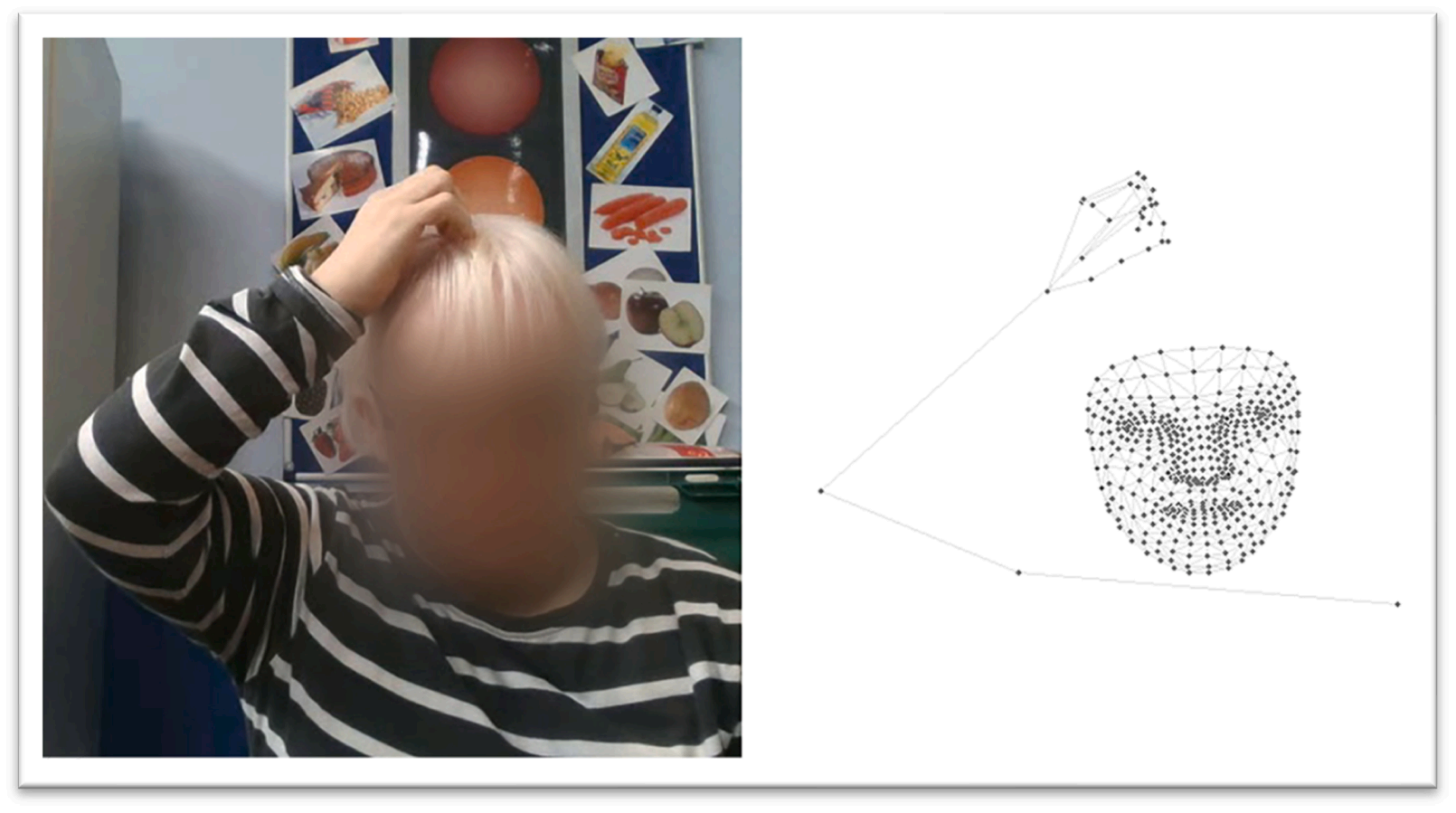
Python’s MediaPipe landmark detection.

**Fig. 9:**
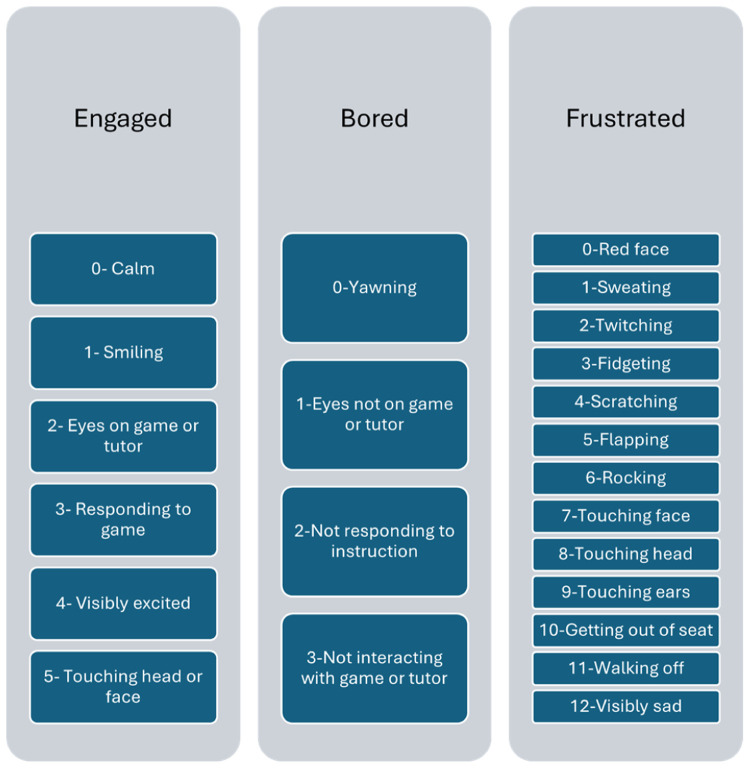
Affective and physiological arousal states defined in observational behavioural checklist.

**Fig. 10:**
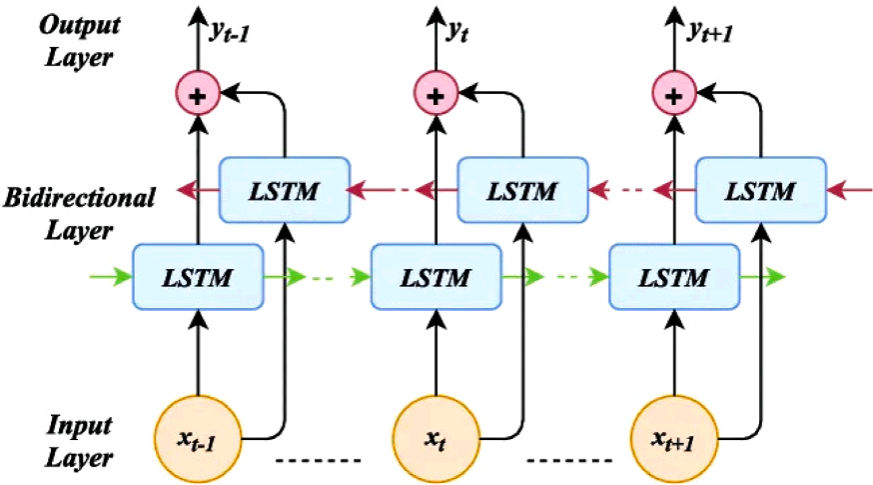
Bi-LSTM Architecture 7.

**Fig. 11:**
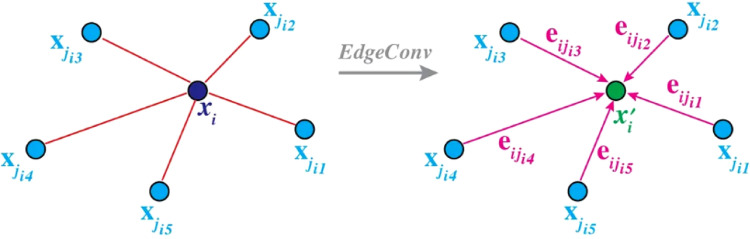
Output of EdgeConv-Source [8].

**Fig. 12:**
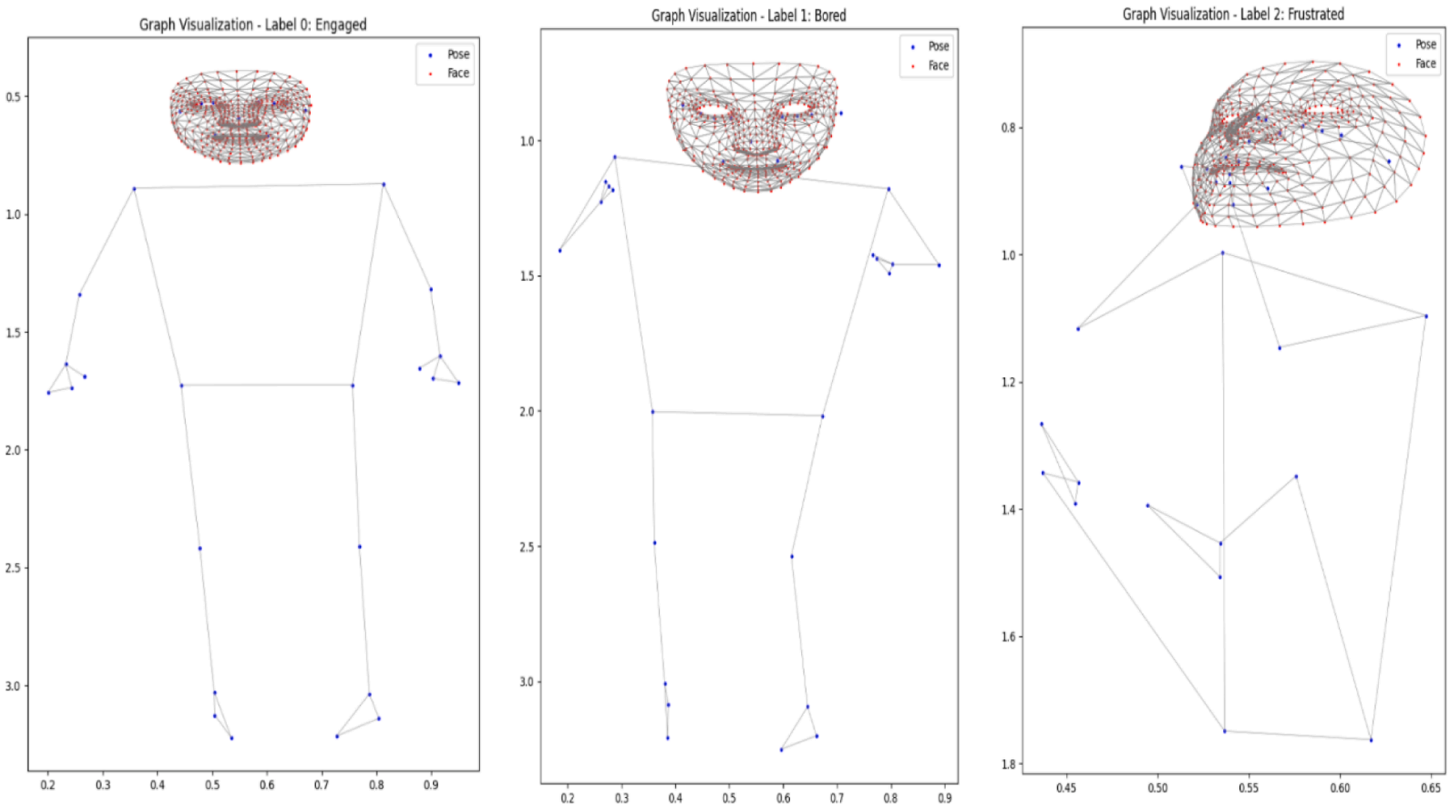
Graph representations of human pose (blue dots) and face landmarks (red dots).

**Fig. 14:**
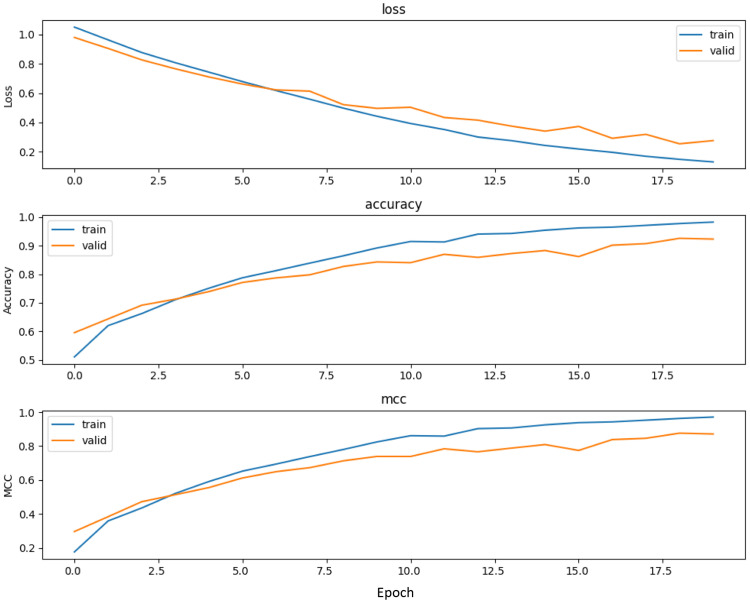
Training metrics for PointCNN+LSTM.

## Method details

This section discusses the end-to-end data processing pipeline used in our study. The first step in our methodology involved collecting raw video data in classroom settings, forming the basis for an anonymised dataset. The dataset was prepared in different stages such as data collection, cleaning, labelling and data presentation. The block diagram below gives an overview of the entire process. Each phase is then explained in detail.

### Video data collection:

Researchers in Computer Science at Nottingham Trent University collaborated with the Nottingham City Council Special Education Needs and Disabilities Team to conduct data collection across five schools in Nottingham. The study focused on children who had a formal diagnosis of Autism, were suspected to be on the Autism spectrum, or were in the process of undergoing diagnostic assessment. Participants were between 5 and 11 years old, representing early- to middle-primary-school age. The aim was to include a diverse sample in terms of support needs, learning environment and behavioural profiles to build a dataset that reflects the broad spectrum of Autism.

Schools were initially approached with a brief overview of the study, including its purpose and what participation would involve. Teachers, who work closely with students daily, helped identify participants for the study. Informed consent was obtained from the parents or legal guardians of these children prior to their involvement in the study. Data were collected via webcam recordings of participants engaged in a series of CPT games. CPT is a neuropsychological test that helps measure a person's sustained and selective attention [9]. It has been widely used to assess attention and response control deficits in individuals with ASC [10].

In total, four games were presented to participants, one at a time, with each increasing in difficulty. Each game had a signal slide containing a target image, a target imitation image and a contrast image, followed by a blank slide shown to the participant. Whenever a participant spotted the target image, they were required to respond by clicking or tapping on an on-screen red button.

Before beginning each session, time was taken to put the participants at ease, during which they were introduced to the game's characters using flashcards and the rules were explained. The game also provided the option to increase or decrease the display times of the signal and blank slides, as well as the number of images on each pattern slide (in the case of the SeekX and SeekAX games). These parameters could be set according to the child's aptitude under observation.

Captura Software [11] was run in the background to record the entire session, simultaneously capturing the screen (gameplay) and the camera feed (participant reactions). Captura sessions were run once for each participant, during which they played the four games in order of increasing difficulty. Depending on the results of each game playthrough, the researcher conducting the session adjusted the settings. During each session, the webcam was positioned to face the participant to capture facial expressions and body movements, enabling labelling of the associated affective states related to learning under varying ambient lighting conditions. Both the screen recordings and video streams were then stitched together using a simple PowerShell script in preparation for the labelling stage.

The research team worked with 38 students (20 males, 17 females, and 1 withdrew) across the participating schools, in a total of 74 sessions, each averaging around 12 min, with some lasting up to 28 min. Whenever a participant appeared tired or asked to stop playing, we anticipated that they would transition into a rumbling stage characterised by subtle behavioural changes. Common indicators included throat clearing, muscle tension, foot tapping, or grimacing. In some instances, associated behaviours were much more intense, such as withdrawing physically or emotionally, or harming themselves or others present in the surrounding environment [12]. During the study, participants were not deliberately stressed; rather, the games served as CPT, allowing natural fluctuations in engagement and self-regulation to occur. This approach enabled us to identify and interpret behavioural markers associated with different phases of learning and attention.

### Video data labelling:

Following the data collection process, data labelling was carried out. An observational behavioural checklist was developed by educational psychologists with expertise in Autism from Nottingham City Council (NCC-UK). This checklist was recreated in the VGG Image Annotator (VIA) software to enable labelling. VIA is a simple, standalone manual annotation software for use with image, audio, and video data, which runs as an offline application in a web browser.

Eight NTU researchers representing diverse ethnic backgrounds and varying levels of experience took part in the video labelling process. Data annotation training was provided by specialists in ASC from NCC-UK, with clear guidelines on how to use the checklist effectively to identify early signals of arousal and engagement in learning (e.g., smiling, calm, yawning, rocking, eyes on or off-screen). To minimise bias and ensure consistency, annotators followed a shared labelling protocol, participated in regular review sessions, and inter-reliability checks were conducted regularly to identify and resolve any discrepancies, thereby ensuring the accuracy and reliability of the annotated behavioural data.

### Data segmentation:

Following completion of the labelling process, the annotated video data were programmatically segmented into shorter clips using the MoviePy Python library [8], based on labels assigned by the trained annotators. Each segment corresponded to a specific labelled affective state (engaged, bored, frustrated), allowing for more precise and manageable units of analysis. The segmentation process was based on the timestamp data generated during annotation to ensure accuracy and consistency. Once segmented, the video clips were securely stored in NTU’s secure datastore, accessible only to authorised personnel involved in the project. All data handling and storage procedures strictly adhered to GDPR regulations and NTU’s data protection policies, ensuring the confidentiality and integrity of participant information. Table 1, Table 2, Table 3, Table 4, Table 5

**Table 1:** Example of a labelling instance.

id:	1_pBzepNT4
**file_list:**	["file:///z:/CombinedVideo/na01_2023–03–21–09–58–50.mp4\r"]
**temporal_segment_start:**	154.209
**temporal_segment_end:**	195.38855
**metadata:**	{"events":"0″,"Tutor in view":"0″,"Engaged":"0,3,2"}

**Table 2:** Architecture design for BiLSTM.

LSTM (Bi-directional, input_dim=1503 → hidden_dim=64)
LSTM (Bi-directional, input_dim=128 → hidden_dim=32)
Linear (input=64 → output=64)
ReLU
Linear (input=64 → output=1)

**Table 3:** Architecture design for EdgeConv-based GNN.

EdgeConv Layer (EdgeConv, in_channels → hidden_channels)
ReLU Activation
EdgeConv Layer (EdgeConv, hidden_channels → hidden_channels)
ReLU Activation
Global Max Pooling
Fully Connected Linear Layer
Activation + Dropout
Fully Connected Linear Layer (hidden_channels → num_classes)

**Table 4:** Architecture desing for PointCNN+LSTM.

PointCNN Layer	Input: (B, T, N, 3) - Point cloud sequence
	Sampling (select P representative points from N input points using rand)
	Finding K nearest neighbhours
	X-Conv
	Output: (B, T, P*C_out) - sequence of frame features
LSTM Layer	Input: Sequence of T frame features
	LSTM - (1, B, lstm_hidden)
	Output: Final hidden state
	Classifier (B → num_classes)

**Table 5:** Training Metrics.

Architecture		Precision	Recall	F-1 Score	Overall Accuracy
**Bi-LSTM**	Engaged	0.91	0.96	0.93	86%
	Bored	0.84	0.85	0.84	
	Frustrated	0.75	0.60	0.67	
**GNN-EdgeConv**	Engaged	0.97	0.96	0.97	94%
	Bored	0.92	0.97	0.95	
	Frustrated	0.91	0.73	0.81	
**PointCNN+LSTM**	Engaged	0.99	0.99	0.99	96%
	Bored	0.98	0.98	0.96	
	Frustrated	0.96	0.95	0.96	

### Extraction of data points using MediaPipe

The segmented video clips were then anonymised while retaining the necessary behavioural information. Data extraction was performed on the segmented video clips using Google’s MediaPipe framework. Mediapipe is Python’s open-source library for performing computer vision inference over images, videos and audio. The Face Landmarker and Pose Landmarker tools were employed to detect and track key facial features and posture features. These tools generated a total of 501 landmark points per frame, comprising 33 pose landmarks and 468 face landmarks, creating a point cloud. A point cloud is represented as a set of 3D points {Pi| i = 1,…,n}, where each point Pi is a vector of its (x,y,z) coordinates referring to its position vector relative to the origin. Each landmark was represented in three-dimensional space: the *x* and *y* coordinates denoted the normalised horizontal and vertical positions (ranging from 0.0 to 1.0), and the *z* coordinate indicated relative depth, with lower values corresponding to points closer to the camera. The resulting data contained no personally identifiable information and could not be linked back to individual participants; hence, it was fully anonymised. Thus, this approach preserves privacy while still allowing for the analysis of affective states related to learning. In Fig. 9, the frame on the left is marked under the label “scratching”, while on the right is the corresponding state represented by the 543 points tracked by Mediapipe [13];Fig. 11, Fig. 10.

### ASC-Emotion: dataset description

The ASC-Emotion dataset [14] presented in this article comprises raw data extracted from video recordings of children with Autism engaged in CPT games. Each frame is represented as a point cloud of 501 three-dimensional coordinates, consisting of 468 facial landmarks and 33 pose landmarks, generated using Python’s MediaPipe library to capture the affective states related to learning and psychological arousal. In total, the dataset contains 587,350 annotated frames derived from 2511 video segments. Each frame is labelled with one of the three primary states i.e. Engaged, Bored and Frustrated, which are further subdivided into specific subclasses to provide a more fine-grained interpretation of the child’s emotional and behavioural condition at a given moment.

This dataset is intended for use in research related to challenging behaviours in children with Autism during classroom learning. Early detection of such behaviours can enable timely and targeted interventions, helping to reduce emotional dysregulation and minimise classroom disruption. To assess the dataset's practical applicability, a series of machine learning models was implemented and is presented in the next section.

## Method validation

The proposed dataset was validated by applying various machine learning models designed to process and analyse 3D point cloud data. 3D data points were fed as input to machine learning models to predict a child's affective state. Each row in the dataset represents a single frame from a video segment and consists of 1503 points (501 3D coordinates), representing facial and pose landmarks extracted using Mediapipe’s holistic model, along with an associated affective state label. The objective was to assess the dataset's utility in capturing meaningful behavioural patterns related to arousal and engagement. To evaluate the model’s performance, standard metrics such as accuracy, precision, recall, and F1-score were employed. The following sections provide a detailed description of each machine learning model used, including its architecture, training approach, and predictive results.

## BiLSTM

Bidirectional Long Short-Term Memory (LSTM) networks are a specific form of Recurrent Neural Networks (RNNs) that process sequences in both forward and backward directions, fully capturing the characteristics of time-series data. Since the point cloud dataset has been extracted from video segments and thus contains sequential information, a BiLSTM architecture can be applied to it.

To standardise the input sequence for model training, a fixed length of 32 frames was used. Since the original sequences varied in length, a padding and truncation strategy was applied. Specifically, for sequences shorter than 32 frames, the last frame was repeated to pad the sequence to the desired length, preserving temporal characteristics and ensuring a uniform input size. After preprocessing, the fixed-length sequences and their corresponding labels were converted into PyTorch tensors for model training.

The model architecture consisted of two bi-LSTM layers with 64 and 32 hidden units, respectively. The output from the final step is then passed to a fully connected layer and ReLU activation, followed by a final classification layer with a softmax output corresponding to three labels (engaged, bored, frustrated).

## Graphical neural network - EdgeConv

The 3D point cloud dataset can be represented as a graph by treating each landmark as a node, connected to other landmarks according to a predefined list [15]. The connection between the two landmarks forms an edge in the graph. This graph-based representation captures the local and global structure of the data, enabling more effective analysis using graph algorithms. A graph neural network (GNN) is a type of neural network specifically designed to operate on data structured as graphs. In particular, EdgeConv is a module designed specifically for point cloud data [16]. It effectively captures the geometric structure while preserving permutation invariance, which is crucial in our case, as the dataset is derived from video segments and is aimed at predicting subjects' affective state. EdgeConv constructs a local neighbourhood graph and applies a convolution-like operation to edges connecting each pair of neighbouring points.

An edge feature is defined as:

(1) $$E_{\mathrm{ij}}=H\left(X_{i},X_{j}\right)$$

Where Xi and Xj are feature vectors of node i and j, while H is a non-linear function with a set of weights. The output of EdgeConv at the i*^th^* node is as follows, where the aggregation is performed using either max pooling or summation:

(2) $$x_{i^{\prime }}=\max_{j:\left(i,j\right)\in E}h_{\Theta }\left(x_{i},x_{j}\right)$$

**Fig. 13:**
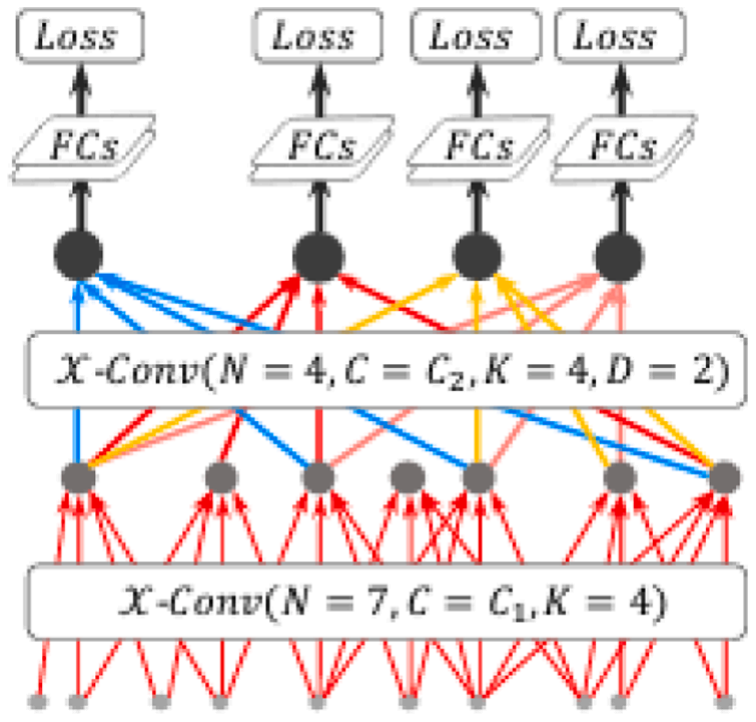
PointCNN architecture for classification.

To prepare the dataset for EdgeConv, facial and pose landmark data were converted into structured graph representations using PyTorch Geometric (Fig. 13). Edges between two nodes (landmarks) were defined using MediaPipe’s pre-defined pose and facemesh topologies [15]. Each labelled sample from the dataset was converted to a data object containing a node, edge and corresponding label. These graphs were serialised and saved for model training and evaluation.

A simple EdgeConv-based neural network was designed to classify the affective states related to learning using the graphs generated from the point cloud dataset. The architecture consists of two stacked EdgeConv layers in which node features are updated by applying a multi-layer perceptron (MLP) to the pairwise difference between a node and its neighbours. After two EdgeConv layers and a ReLU activation, global max pooling is applied to obtain a graph-level feature vector. This is followed by a fully connected MLP with dropout for classifying the target classes, i.e., engaged, bored, and frustrated. Thus, the model learns the spatial relationship of a landmark relative to its neighbours and uses this information for classification.

## PointCNN±LSTM

PointCNN is a deep learning framework designed for feature learning from point clouds [17]. The model processes input data $F_{1}=\left(p_{1i}f_{1i}\right):i=1,2,\ldots ,N_{1}$, which $p_{1i}:p_{1i}\in R\text{dim}$ is a set of points associated with features, and applies the X-conv operator to it. For each point P in the dataset, X-conv identifies its K nearest neighbours and their corresponding features, producing a new feature vector F_p_ that represents the aggregated information at point P, effectively capturing local spatial patterns. The operation of the X-conv function can be summarised by Eq. (3), where MLP is applied individually to each point, as in PointNet [18].

(3) $$F_{p}=X-Conv\left(K,p,P,F\right)=Conv\left(K\;MLP\left(P-p\right)\left[MLP\left(P-p\right)\;F\right]\right)$$

Each row in the dataset corresponds to a single video frame, with multiple rows forming a sequence. To model the spatiotemporal pattern in the dataset, a two-stage learning architecture was employed, i.e. PointCNN+LSTM. PointCNN was applied independently to each frame to extract a feature vector capturing the geometric structure of the point cloud. These frame-level features are then fed into an LSTM network as a sequence to capture the temporal dependencies across frames. The hidden final state of the LSTM was passed to a classification head to predict the target label, representing the affective states associated with learning.

## Validation outcomes and discussion

To validate the curated ASC-Emotion dataset, three deep learning architectures were trained and evaluated. Their performances were assessed using standard classification metrics, precision, recall and F-1 score.

The Bi-LSTM model was trained on 1757 samples with 377 samples for testing and validation. Each sample contained 32 frames to capture the temporal pattern. The model was implemented with Adam Optimizer with a learning rate of 0.001 and cross-entropy loss as the objective function. A learning rate scheduler (ReduceLROnPlateau) reduced the learning rate by a factor of 0.5 upon stagnation in validation loss, with a patience of three epochs. Early stopping with a patience of seven epochs was employed to prevent overfitting. Training plateaued at 69 epochs with a validation accuracy of 87%. The model exhibited strong performance on the test set as well with an overall accuracy of 86%. It detected *Engaged* (F1=0.93) and *Bored* (F1=0.84) states well; however, it showed a substantial drop in accuracy of the *Frustrated* state, with a F1 score of 0.67.

The GNN-EdgeConv model was trained on approximately 411,145 samples, each of which is a graph representing a single frame from the dataset. The validation and test samples were around 45,100. Each batch contained 32 graphs, and the model was optimised using the Adam optimizer (learning rate = 1 × 10⁻⁴) with cross-entropy loss for three-class classification. Training plateaued after 50 epochs. This model achieved more balanced results, with an overall accuracy of 94% across all three affective states related to learning. The model demonstrated strong performance on the *Engaged* (F1=0.97) state, while most misclassifications occurred between the *Bored* (F1=0.95) and *Frustrated* (F1=0.81) states. This indicates that the model is struggling to differentiate between the bored and frustrated states, likely due to the overlapping of behavioural cues.

The PointCNN+LSTM architecture processed sequences of 32 3D point clouds. PointCNN captured geometric patterns, followed by an LSTM layer for temporal modelling. Each frame is encoded using RandPointCNN, with XYZ coordinates as features. Frame embeddings are then flattened and passed to an LSTM architecture for temporal aggregation, followed by a fully connected layer for classification. The model was trained using the Adam Optimiser with a learning rate of 1 × 10⁻⁴ for 20 epochs. This model outperformed the other models, achieving the highest overall accuracy of 96% and F1 scores of *0.99 for Engaged*, 0.96 for *Bored,* and 0.96 for *Frustrated*. Similarly, the Matthews Correlation Coefficient (MCC) increased throughout training, reaching 0.97 for training and 0.88 for validation, indicating a strong correlation between predictions and true labels across all classes. The parallel improvement of training and validation metrics suggests stable generalisation and effective learning (Figure 15).

The Bi-LSTM architecture demonstrates an efficient temporal modelling along with very low computational cost; however, it fails to learn geometric patterns, which is an important characteristic of our dataset. This limitation is also evident in the training results. On the other hand, GNN-EdgeConv captures strong spatial features but does not account for temporal patterns, as each frame is processed as a separate graph. PointCNN+LSTM achieves balanced spatial-temporal modelling, highlighting the effectiveness of this hybrid approach, at the expense of computational efficiency. Additionally, incorporating attention mechanisms in future work could enable the network to dynamically focus on the most emotion-relevant regions of the point cloud over time.

The field of affective computing has raised many concerns regarding privacy preservation [19]. As a key contribution, this study introduces a novel, privacy-preserving dataset that captures diverse affective states related to learning and physiological arousal exhibited by children with Autism during classroom activities. The dataset is completely anonymised, while retaining essential facial and pose landmark information for analysis. The cloud points dataset is then further used to validate its utility using three different machine-learning architectures. This work provides both a valuable resource and a methodological foundation for advancing privacy-aware affective computing research in sensitive, real-world settings.

## Conclusion and future work

ASC-Emotion is a novel dataset specifically curated to study learning behaviours in children with Autism in classroom environments. This dataset enables early detection of emotional dysregulation events, supporting the timely introduction of evidence-based well-being interventions to minimise their frequency and intensity. It will not only enhance the learning environment by minimising classroom disruption, but also foster more positive perceptions and understanding of Autism.

The current study focuses on optimising classification accuracy for validating a dataset for a privacy-preserving approach to meltdown and engagement detection in children with Autism during classroom learning. An important extension of this work is to make these models interpretable to humans, i.e. explainable AI (XAI). XAI approaches will not only provide insights for the decision-making process to parents, teachers and caregivers of autistic children but also align with ethical and practical considerations in affective computing, where transparent and interpretable predictions are essential for trust, user acceptance and deployment in sensitive domains, such as with people with Autism and learning disabilities in educational environments and in wider contexts.

## Limitations

Autism is a spectrum; each child with ASC has a different diagnosis and exhibits a different behavioural pattern. This makes the dataset heterogeneous; however, it also lends diversity. Since the data is collected from different schools in multiple sessions, videos vary in quality, such as lighting and positioning of the webcam, which in some cases makes it difficult to detect landmarks. Moreover, children with Autism have delayed cognitive functions, due to which they may struggle to comprehend and recall new information. Therefore, with many students, it was difficult to help them understand what was expected from them and follow instructions to perform the experiment. Also, if at any point during the data collection session, a child started to experience sensory difficulties, the session was paused.

## Ethics statements

Work carried out for the collection of this dataset has been carried out in accordance with The Code of Ethics of the World Medical Association (Declaration of Helsinki) for experiments involving humans. The data collected during the experiment were in accordance with the ethics statement granted for the EU Erasmus AI-TOP project by the NTU Non-Invasive Ethics Committee (application id: 1553,053), which states that all the data collected will follow established ethical and legal practices and all information about participants and school/organisation will be handled confidentially. Informed consent was taken from parents, teachers and caregivers of all children participating, and all identifying information collected during the study was kept strictly confidential. Any information which contained the names and identities of participants was removed prior to any analysis or publication of results.

## CRediT author statement

**Zakia Batool Turabee** – Conceptualization, Methodology, Software, Investigation, Data Curation, Writing - Original Draft **David J. Brown** – Conceptualization, Methodology, Supervision, Writing - Review & Editing **Mufti Mahmud** – Conceptualization, Curation, Supervision, Writing - Review & Editing **Andreas Oikonomou** – Supervision, Writing - Review & Editing **Muhammad Arifur Rahman** - Investigation, Data Curation, Writing - Review & Editing **Andrew Burton** – Software, Investigation, Data Curation, Writing - Review & Editing **Nicholas Shopland** – Software, Investigation, Data Curation, Writing - Review & Editing.

## Acknowledgments

This research was co-funded by the Erasmus+ Programme of the European Union in the project AI-TOP (AI-TOP - 2020-1-UK01-KA201-079,167).

The authors would like to thank Nottingham City Council Autism Department for providing their domain expertise. We would also like to express gratitude to parents of children who gave consent for their children to take part in the experiments.

Supplementary material *and/or* additional information [OPTIONAL]

## Declaration of competing interest

The authors declare that they have no known competing financial interests or personal relationships that could have appeared to influence the work reported in this paper.

## Data Availability

The data is publicaly available and doi is provided in the manuscript.
